# Reporting ethical aspects in published research articles in the Indian Journal of Psychiatry

**DOI:** 10.4103/0019-5545.44902

**Published:** 2009

**Authors:** Santosh K. Chaturvedi, B. S. Somashekar

**Affiliations:** Department of Psychiatry, National Institute of Mental health and Neurosciences, Bangalore, India; 1 Coventry and Warwickshire NHS Partnership Trust, The Caludon Centre, Clifford Bridge Road, Coventry, CV2 2TE, UK

**Keywords:** Ethical approval, Indian Journal of Psychiatry, informed consent, psychiatric research

## Abstract

**Background:**

Reporting of informed consent and ethical approval are important aspects of published papers which indicate the knowledge and sensitivity about ethical aspects of research by the researchers.

**Materials and Methods:**

This study reports description of informed consent and ethical approval in the published psychiatric research in the main journal of psychiatry in India. All original research articles (n=157) published in the Indian Journal of Psychiatry in the years 2000, 2003 to 2007 were included.

**Results:**

Informed consent was mentioned in 51% of studies in 2000, which gradually rose to 82% by the year 2007. Ethics committee approvals were mentioned in 2% of studies in 2000, and 25% of reports in 2007. Consent was reported to be written in only 40%, content of the consent forms was mentioned in 17%, and the language of consent form was reported in 3% of the studies where consent was reported.

**Conclusions:**

Regulation of ethical principles and formulation of necessary guidelines or rules for research as well as for publications are necessary and desirable to ensure the safety of participants and good quality of research.

## INTRODUCTION

Almost all nations around the world are familiar with the concept of informed consent and ethical approvals, though the standards of their understanding and implementation differ. The system is paternalistic in some countries where the clinician takes decisions for patients and diametrically opposite, self-deterministic in others, which emphasize patient autonomy. Other countries may lie somewhere along the spectrum and appear heading towards the transparent model. It is a well-recognized principle in medical ethics that the consent of a patient should be obtained before performing any procedure whatsoever, whether it be invasive or noninvasive, for the purpose of research or for treatment. It has also been a legal requirement to conduct research. Informed consent is meaningful only when potential research subjects assess the relevant risk and benefits of proposed intervention and then voluntarily gives authorization to proceed.

Informed consent assumes more importance in psychiatric research due to issues related to competence of a person with psychiatric disorder to give consent, validity of the consent given by a patient with lack of insight or impaired judgment, and the proxy consent given by a relative or caregiver. Finally, one may question if it really is possible to obtain free and informed consent from a psychiatric patient with impaired judgment?

There is plenty of international literature on concerns regarding informed consent in psychiatric research, whether informed consent (for research or treatment) can be obtained from psychiatric patients, and how competency is established, however, there are very few reports from our country on different aspects of informed consent[1–5] competence.[6]

There has been a steady growth of psychiatric research in India. The leading journals all over the world proscribe a strict guideline to be followed in the conduct and publication of research. There are no studies, which have examined the issue of consent and ethical approval in the published psychiatric research in India. In this study, we attempted to examine whether informed consent and ethical approval were reported in the published psychiatric research in the Indian Journal of Psychiatry (IJP), the official journal published by Indian Psychiatric Society

## MATERIALS AND METHODS

All original research articles published in the Indian Journal of Psychiatry in the years 2000, 2003 to 2007 were included in the study. Research articles, which warrant consent such as drug trials, trials involving electroconvulsive therapy, studies involving invasive procedures and clinical interviews undertaken for research (not part of routine clinical assessment) were included in the assessment. Retrospective chart reviews and case reports were excluded, as it is still not mandatory to obtain consent if the identity is not disclosed. Editorials, reviews, letters to the editor and book reviews were excluded, as consent was irrelevant. The information collected was whether consent obtained or not, the procedure of obtaining consent; adequate description of the content of consent form, the language in which it was written etc; was the consent informed or not; adequate information about the protocol of research was provided or not, whether the consent was written or oral, and who provided the consent. Information about ethical committee approval was also noted from each paper published.

In addition, the information regarding the study such as nature of the study, nature of the intervention and the procedure followed in the study - invasive or noninvasive - was also obtained. Collection of blood and radiological investigations other than for routine clinical requirements were considered as invasive. Procedure such as EEG, interviews and interview-based surveys were considered as noninvasive. The data was computed and different aspects of consent were analyzed.

[Ethical aspects: Not applicable; Review of published literature; study does not involve human subjects or animals]

## RESULTS

There were a total of 157 published studies for which consent and ethical approval should have been obtained. None of case reports obtained consent, however, they did not reveal the identity of the patient. Informed consent was mentioned in 51% of studies in 2000, which gradually rose to 82% in the year 2007 [Table 1]. In 2004 and 2007, 82% of reports had a mention of consent, however, the number of reports published in these years were fewer than other years.

**Table 1:** Informed consent and ethics approval

Year and number of studies	2000 n=37 (%)	2003 n=25(%)	2004 n=17(%)	2005 n=33(%)	2006 n=29(%)	2007 n=16(%)	Total (%)
Informed consent obtained	19 (51)	15 (60)	14 (82)	21 (63)	18 (62)	13 (81)	64
Content of consent form mentioned	1 (3)	4 (16)	5 (29)	2 (6)	3 (10)	2 (13)	17*
Language of consent form or process	0 (0)	0 (0)	1 (6)	0 (0)	1 (3)	1 (7)	03*
Written consent mentioned	7 (19)	7 (28)	4 (24)	8 (24)	9 (31)	5 (31)	40*
Ethics committee approval obtained	2 (5)	2 (8)	3 (18)	5 (15)	8 (28)	4 (25)	15

* Percent of those reports with informed consent obtained

The ethical committee approval was sought only by studies conducted at major institutes. The ethics committee approvals were mentioned in 2% of studies in 2000, which rose to 28% in the year 2006 and 25% of reports in 2007. None of the studies described the procedure of obtaining the consent. The consent sought was reported to be written in 40% of the studies reporting consent being sought. In other studies, it is not clear if the consent was verbal or written. The content of the consent forms has been mentioned briefly in a small proportion of reports (17%) and the language of consent form has been specified only occasionally (3%).

Consent was obtained more often in studies involving drug trials and invasive procedure than in studies with interviews or scales. Consent was written, informed, and approved by the local ethical committee when pharmaceutical company funded the studies. In a couple of population based surveys, consent and approval was also sought from the local community leaders.

## DISCUSSION

There has been a gradual rise in the proportion of studies where consent has been reported. However, there is a great scope for improvement in seeking and reporting the details of the consent. Ethical approval is still not reported for almost 75% of the reports, which is a cause for concern. This may be due to lack of availability of ethics committees to the researchers. It may also be due to the perception of researchers that interview forms and scales can be administered as a part of the study with informed consent alone, and without need of approval of an ethics committee. Many institutes insist on an ethical approval only for funded projects and drug trials, and not other non-funded, interview or assessment based studies.

It is evident from the results that more than half of the published studies did not mention even a single word on consent. Although consent was obtained more often in drug trials and studies with invasive procedures but the consent was inadequately described. Written informed consent and ethical committee approval for drug trial funded by pharmaceutical company were reported in most trials.

The reasons for the gross inadequacy of obtaining consent are difficult to explain. This may not indicate a deliberate attempt not to obtain consent on the part of clinician or researcher. Possible speculations include absence of monitoring, illiteracy of the patients, considering doctors as best judges by the patient and their relatives.[4] In India and probably in most developing countries, a doctor has to work as a clinician, researcher and as a teacher. The support from the government for the research activities is minimal from governmental organizations such as Indian Council for Medical Research, which has also laid down detailed guidelines for ethical research.[7] The findings also indicate the absence of absolute requirement of reporting informed consent and ethical approval by the Journal. Inadequate ethical self-regulation could hamper autonomy of researchers, quality of research and violate the rights of participants. In general, it could lower the respect for and social commitments of the health research in general and psychiatric research in particular. The findings should not be interpreted as a lack of ethical principles being followed by the researchers, or that consent, verbal or written, was not being sought. The researchers, authors, and editors need to be sensitive and responsible towards adequate reporting of the ethical and consenting process followed. Similar findings were reported in a recent study reporting ethical approval and consent in two pediatrics journals from India. Ethical approval was mentioned in 29%, and informed consent in 49% of the published reports.[8] Reporting of informed consent and ethical approval are important aspects of published papers which indicate the knowledge and sensitivity about ethical aspects of research by the researchers as well as safeguarding the interests of the participants. This report does not discuss important information in terms of an overall view of Indian ethics in research system and regulation, which would require a different type of methodology, but has been highlighted by other essays.[1,2] This report raises the issue on the conduct of research and thus the question of transparency of reporting and also of journal guidelines. In this context, it may be worthwhile for the editor of the IJP to be a co-signatory to or support the recent call by editors of Indian biomedical journals for registering clinical trials.[9] The Journal already asks its reviewers about ethical aspects during the reviewing process; it could be more explicit in asking the reviewer to confirm if information on review by an ethics committee has been obtained and informed consent have been described or not. The journal could ask all authors to clearly mention this aspect in the section on Materials and Methods, maybe before describing the statistical methods used or at the end of the Methods section, or in a box at the end of the article, along with disclosure of competing interests or conflict of interests. Even for Case Reports reporting of written informed consent from the competent subject and /or key relative should be insisted upon. Minimum ethical aspects which need to be mentioned could be clearance by an ethics committee, informed consent sought (written or verbal, language/s used, from patient or relative), number of subjects who refused consent, and competence and confidentiality issues where ever applicable. Guidelines for a standard informed consent form have been provided by the ICMR,[7] and are also available on the internet from other sources.

Clinician researchers whose Institutes do not have ethics committees and those who are not based in academic Institutes can approach Independent Ethics Committees, which are private organizations and offer to scrutinize ethical aspects based on ICMR guidelines,[7] ICH guidelines for Good Clinical Practice,[10] and the Helsinki Declaration[11] for a fee. Some such facilities and contract research organizations can be found on the internet, if one searches using key words ‘Independent Ethics Committees, India’. Similarly, there are private Scientific Review Committees, which can help such clinical researchers. Academic institutes need to have Institutional Review Board [IRB], whereas those not in such Institutes could constitute their own Independent Ethics Committees [IEC] on the guidelines, terms and conditions and method on the formation and functioning of ethics committees provided in the ICMR[7] guidelines, the ICH GCP,[10] WHO's operational guidelines for ethics committees that review biomedical research[12] or the Good Clinical Practice guidelines of the Directorate General Health Services, DGHS, India.[13] It is the researchers' responsibility to know which projects require ethical clearance[7,14] and this adds to the credibility of research and ascertains good research practice.

## CONCLUSIONS

Informed consent was not reported in about 50% of the published studies in the 2000 about 20% of studies in 2007. It was inadequate and unwritten in majority of the studies. However, there is a gradual improvement in reporting of consenting process and ethical approvals over the years. Regulation of ethical principles and formulation of necessary guidelines/rules for research as well as for publications are necessary and desirable to ensure the safety of participants and good quality of research.
